# Acupuncture decreased the risk of coronary heart disease in patients with fibromyalgia in Taiwan: a nationwide matched cohort study

**DOI:** 10.1186/s13075-017-1239-7

**Published:** 2017-02-28

**Authors:** Mei-Yao Wu, Ming-Cheng Huang, Jen-Huai Chiang, Mao-Feng Sun, Yu-Chen Lee, Hung-Rong Yen

**Affiliations:** 1 Research Center for Traditional Chinese Medicine, Department of Medical Research, China Medical University Hospital, 2 Yude Road, North District, Taichung, Taiwan; 2 Department of Chinese Medicine, China Medical University Hospital, 2 Yude Road, North District, Taichung, Taiwan; 3 Graduate Institute of Chinese Medicine, School of Chinese Medicine, College of Chinese Medicine, China Medical University, Taichung, Taiwan; 4 Health Data Management Office, China Medical University Hospital, 2 Yude Road, North District, Taichung, Taiwan; 5 Research Center for Chinese Medicine & Acupuncture, China Medical University, Taichung, Taiwan; 6 Graduate Institute of Acupuncture Science, College of Chinese Medicine, China Medical University, Taichung, Taiwan

**Keywords:** Acupuncture, Coronary heart disease, Fibromyalgia, National Health Insurance Research Database, Taiwan

## Abstract

**Background:**

The aim of this study was to understand whether acupuncture can decrease the risk of coronary heart disease (CHD) in patients with fibromyalgia.

**Methods:**

Using data from the Taiwanese National Health Insurance Research Database, we performed a propensity score-matched cohort study to analyze patients with fibromyalgia diagnosed between 1 January 2000 and 31 December 2010. Patients who received acupuncture treatment, beginning with their initial date of fibromyalgia diagnosis and extending to 31 December 2010, were regarded as the acupuncture cohort. The no-acupuncture cohort comprised patients who never received acupuncture through 31 December 2010. A Cox regression model was used to adjust for age, sex, comorbidities, and drugs used. The HRs of the acupuncture and no-acupuncture cohorts were compared.

**Results:**

After performing a 1:1 propensity score match, 58,899 patients in both cohorts were identified. Baseline characteristics were similar in both cohorts. The cumulative incidence of CHD was significantly lower in the acupuncture cohort (log-rank test, *p* < 0.001). In the follow-up period, 4389 patients in the acupuncture cohort (17.44 per 1000 person-years) and 8133 patients in the no-acupuncture cohort (38.36 per 1000 person-years) developed CHD (adjusted HR 0.43, 95% CI 0.41–0.45). The beneficial effect of acupuncture on the incidence of CHD was independent of age, sex, comorbidities, and statins used.

**Conclusions:**

Our study confirmed that acupuncture reduced the risk of CHD in patients with fibromyalgia in Taiwan. Further clinical and mechanistic studies are warranted.

## Background

Fibromyalgia, characterized by chronic widespread pain, commonly presents with associated symptoms such as fatigue, sleep disturbance, depression, cognitive dysfunction, and headache [1, 2]. The American College of Rheumatology published the criteria for fibromyalgia, which were modified in 2010 [3]. The global mean prevalence of fibromyalgia in the general population was 2.7%, and the female-to-male ratio was 3:1 [4]. The quality of life (QOL) of patients with fibromyalgia is always impaired, and about one-third of patients have difficulties in performing the activities of daily living. The high incidence of comorbidities is another health care problem. Researchers in previous studies reported that patients with fibromyalgia in the United States were two to seven times more likely to have comorbidities [5]. According to the U.S. National Health Interview Survey, myocardial infarction occurred more than twice as often in patients with fibromyalgia as in patients without fibromyalgia [6]. In Korea, chronic emotional stress in postmenopausal women with fibromyalgia impaired myocardial function [7]. In Taiwan, a population-based cohort study revealed that the risk of coronary heart disease (CHD) in patients with fibromyalgia was 47% higher than in the general population [8].

Current treatment strategies for fibromyalgia include pharmacological and nonpharmacological methods. Pregabalin, duloxetine, and milnacipran are U.S. Food and Drug Administration (FDA)-approved drugs for treating patients with fibromyalgia, but the efficacy of these drugs has been questioned because of the small sample sizes in clinical trials [9]. Nonpharmacological treatments of fibromyalgia include acupuncture, massage, and exercise. Because the pathological mechanisms of fibromyalgia are not yet fully understood, current treatments are focused on attenuating symptoms and improving QOL.

Animal studies and clinical trials revealed that acupuncture has a beneficial effect in the treatment of fibromyalgia [10, 11]. However, there is no long-term follow-up study showing whether acupuncture can decrease the risk of CHD in patients with fibromyalgia. The Taiwanese National Health Insurance Research Database (NHIRD) provides information on the entire population of Taiwan with long-term follow-up data for interventions. The advantage of using this database is that it prevents sampling bias [12]. The National Health Insurance (NHI) program was implemented in 1995 in Taiwan by the National Health Insurance Administration (NHIA). Traditional Chinese medicine (TCM) services have been reimbursed through the NHI program since 1996 [13]. The use of TCM services, including herbal medicine and acupuncture, is common in Taiwan for the treatment of many kinds of disease [14–17]. To understand whether acupuncture can decrease the risk of CHD in patients with fibromyalgia, we investigated a randomly selected sample of 1 million people enrolled in the NHIRD from 2000 to 2010.

## Methods

### Data sources

We conducted a nationwide, population-based, 1:1 propensity score-matched cohort study by analyzing data derived from the NHIRD. The data source of our study was the Longitudinal Health Insurance Database 2000, which contains all the original claims data of 1 million beneficiaries randomly sampled from the registry of all beneficiaries in 2000. The sampled patients exhibited no significant difference in age, sex, birth year, or average insured payroll-related costs from the general population. The International Classification of Diseases, Ninth Revision, Clinical Modification (ICD-9-CM), codes were used for diagnoses. Because the NHIRD contains identified secondary data for research, the present study was waived from the need for informed consent. This study was approved by the research ethics committee of China Medical University and Hospital (CMUH104-REC2-115).

### Study cohort identification

**Fig. 1:**
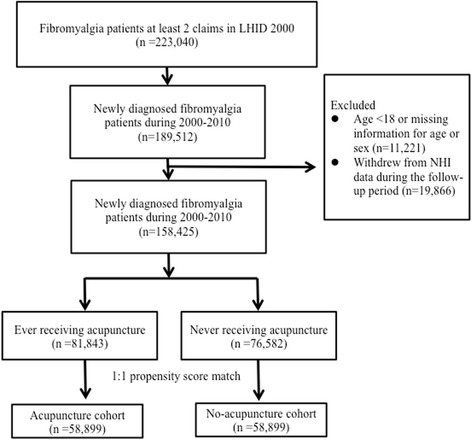
Study population flowchart. After the exclusion process, we identified 158,425 patients with fibromyalgia newly diagnosed between 2000 and 2010. After matching 1:1 by sex, age, comorbidities, and drugs used, the acupuncture cohort and no-acupuncture cohorts each contained 58,899 patients. *LHID 2000* Longitudinal Health Insurance Database 2000, *NHI* National Health Insurance

Patients newly diagnosed with fibromyalgia from 1 January 2000 to 31 December 2010 were identified (Fig. 1). To be included, patients with fibromyalgia should have had at least two ambulatory or inpatient claims with the diagnosis of ICD-9-CM codes 729.0 and 729.1. The exclusion criteria included age younger than 18 years, incomplete information on age and sex, and withdrawal from the NHIRD during the follow-up period. Patients who received acupuncture from their initial diagnosis of fibromyalgia to 31 December 2010 were identified as the acupuncture cohort. Propensity score approaches were used to reduce confounding by the indication of acupuncture treatment. We used a 1:1 propensity score match by sex, age (per 5 years), comorbidities, drugs used, and the year in which fibromyalgia was diagnosed through multiple logistic regression analysis. Ultimately, equal numbers of patients in the acupuncture and no-acupuncture cohorts were analyzed in this study. The immortal time of acupuncture cohort was the period between the first time of receiving acupuncture and the date of initial diagnosis with fibromyalgia. The claims data for both cohorts were assessed from the initial diagnosis date to 31 December 2011 (the end of this study).

### Covariate assessment

Sociodemographic factors included age and sex. Patients were divided into three groups according to their age (18–39 years, 40–59 years, and older than 60 years). Baseline comorbidities were considered present if ICD-9-CM codes appeared two or more times in outpatient or inpatient claims before the initial diagnosis of fibromyalgia, which included diabetes mellitus (DM; ICD-9-CM code 250), hypertension (ICD-9-CM codes 401–405), hyperlipidemia (ICD-9-CM code 272), congestive heart failure (ICD-9-CM codes 402.01, 402.11, 402.91, 404.01, 404.03, 404.11, 404.13, 404.91, 404.93, and 428.0), stroke (ICD-9-CM codes 430–438), depression (ICD-9-CM codes 296.2–296.3, 300.4, and 311), anxiety (ICD-9-CM codes 300.0, 300.2, 300.3, 308.3, and 308.91), alcoholism or alcohol-related disorders (ICD-9-CM codes 291, 303, 305.00–305.03, 790.3, and V11.3), tobacco dependence (ICD-9-CM code 305.1), and obesity (ICD-9-CM codes 278 and A183).

### Types of acupuncture and disease categories in the acupuncture cohort

We analyzed the acupuncture type that patients received by the treatment codes, which included manual acupuncture of TCM type (B41, B42, B45, B46, B80-B84, B90–B94, P27041, P31103, P32103, and P33031) and electroacupuncture (B43, B44, B86–B89, and P33032). Disease categories for which patients with fibromyalgia received acupuncture were identified by the ICD-9-CM codes. More than one ICD-9-CM code may be recorded when patients receive acupuncture, so the total numbers of patients in all categories were more than the number of patients in the acupuncture cohort.

### Statistical analyses

We compared the baseline characteristics of the acupuncture and no-acupuncture cohorts using standardized mean differences. Standardized mean differences with less than 0.1 SD indicated a negligible difference in mean values or proportions between the two cohorts. The HR and 95% CI were calculated for each variable by Cox proportional hazards regression. The difference between the two cohorts in the development of CHD was estimated using the Kaplan-Meier method and the log-rank test.

Statistical analysis was performed and figures were created using SAS 9.4 (SAS Institute, Cary, NC, USA) and R software (R Foundation for Statistical Computing, Vienna, Austria). *p* < 0.05 in two-tailed tests indicated statistical significance.

## Results

Using the NHIRD, we identified 189,512 patients with fibromyalgia newly diagnosed between 1 January 2000 and 31 December 2010 (Fig. 1). After excluding patients with missing information or lacking follow-up, 158,425 patients were included. Among included patients, 81,843 received acupuncture and 76,582 patients never received acupuncture from the initial fibromyalgia diagnosis date to 31 December 2010. To minimize the differences in basic data between these two cohorts, we used a 1:1 propensity score match to randomly select 58,899 patients each for the acupuncture and no-acupuncture cohorts.

The baseline characteristics of both cohorts are shown in Table 1, and they were similar in sex, age, comorbidities, and drugs used. The proportion of women was higher than men in both cohorts, and the dominant age group was 40–59 years. The most common comorbidity was hypertension, which was present in more than 20% of patients. Among the patients with fibromyalgia, 17% had hyperlipidemia, 11% had anxiety, 10.6% had DM, 6.9% had stroke, 4.9% had depression, and 1% had congestive heart failure. The proportions of patients diagnosed with alcoholism or alcohol-related disorders, tobacco dependence, and obesity were similar in both cohorts. Almost all patients in both cohorts used nonsteroidal anti-inflammatory drugs (NSAIDs), and approximately 67% of patients used oral steroids. About 13.5% of patients took statins for hyperlipidemia in the no-acupuncture and acupuncture cohorts. The mean duration between the initial diagnosis of fibromyalgia and the first time receiving acupuncture was approximately 919 days. The average number of acupuncture visits was 7.45. Most patients (85%) received manual acupuncture of TCM type, and 3.6% patients received electroacupuncture. About 10.7% of patients received both types of treatment.

**Table 1:** Characteristics of patients with fibromyalgia in the present study

Variable	No acupuncture (*n* = 58,899)		Acupuncture (*n* = 58,899)		Standardized mean difference
	*n*	%	*n*	%	
Sex					
Female	33,331	56.59	33,447	56.79	0.004
Male	25,568	43.41	25,452	43.21	
Age group, years					
18–39	24,487	41.57	23,572	40.02	0.032
40–59	27,481	46.66	29,748	50.51	0.077
≥60	6931	11.77	5579	9.47	0.075
Mean (±SD), years	44.57 (15.43)		44.28 (14.44)		0.02
Baseline comorbidities					
Diabetes mellitus	6252	10.61	6098	10.35	0.009
Hypertension	12,457	21.15	12,036	20.43	0.018
Hyperlipidemia	10,097	17.14	10,034	17.04	0.003
Congestive heart failure	569	0.97	508	0.86	0.011
Cerebrovascular diseases	4044	6.87	3786	6.43	0.018
Depression	2863	4.86	2840	4.82	0.002
Anxiety	6551	11.12	6600	11.21	0.003
Alcoholism or alcohol-related disorders	311	0.53	316	0.54	0.001
Tobacco dependence	289	0.49	270	0.46	0.005
Obesity	439	0.75	427	0.72	0.002
Drugs used					
Oral steroids	39466	67.01	39495	67.06	0.001
NSAIDs	57613	97.82	57734	98.02	0.014
Statins	7971	13.53	7937	13.48	0.002
Types of acupuncture					
Manual acupuncture of TCM type	–	–	50,470	85.69	
Electroacupuncture	–	–	2142	3.64	
Combination of manual acupuncture and electroacupuncture			6287	10.67	
Duration between the initial diagnosis to the first acupuncture treatment, days, mean (median)	–		919 (574)		
Acupuncture visits, mean	–		7.45		
Follow-up duration, years, mean (median)	3.60 (3.02)		4.27 (3.79)		

*NSAIDs* Nonsteroidal anti-inflammatory drugs, *TCM* Traditional Chinese medicine

During the follow-up period, there were 12,522 patients included in our study who developed CHD (Table 2). The incidence of CHD in patients with fibromyalgia increased depending on age (adjusted HRs 4.24 and 6.24 in 40–59 years and ≥60 years age groups, respectively). Patients with fibromyalgia with comorbidities also developed CHD more easily than patients without comorbidities. Overall, the incidence of CHD was significantly lower in the acupuncture cohort than in the no-acupuncture cohort (adjusted HR 0.43, 95% CI 0.41–0.45).

**Table 2:** Hazard ratios of coronary heart disease associated with acupuncture and covariates among patients with fibromyalgia

Variable	Number of events (*n* = 12,522)	Crude HR	(95% CI)	*p* Value	Adjusted HR	(95% CI)	*p* Value
Received acupuncture							
No	8133	1.00	Reference		1.00	Reference	
Yes	4389	0.48	(0.46–0.5)	<0.0001	0.43	(0.41–0.45)	<0.0001
Sex							
Female	7138	1.00	Reference		1.00	Reference	
Male	5384	0.98	(0.95–1.02)	0.3907	1.03	(0.99–1.07)	0.1072
Age group, years							
18–39	1248	1.00	Reference		1.00	Reference	
40–59	7946	5.24	(4.94–5.56)	<0.0001	4.24	(3.99–4.51)	<0.0001
≥60	3328	11.33	(10.62–12.1)	<0.0001	6.24	(5.81–6.71)	<0.0001
Baseline comorbidities (reference = nonsite comorbidity)							
Diabetes mellitus	2994	2.9	(2.78–3.02)	<0.0001	1.37	(1.31–1.44)	<0.0001
Hypertension	6188	4.03	(3.89–4.18)	<0.0001	2.20	(2.11–2.3)	<0.0001
Hyperlipidemia	4452	2.88	(2.78–2.99)	<0.0001	1.63	(1.56–1.7)	<0.0001
Congestive heart failure	298	3.19	(2.85–3.58)	<0.0001	1.17	(1.04–1.32)	0.0078
Cerebrovascular diseases	1966	2.81	(2.68–2.95)	<0.0001	1.25	(1.19–1.32)	<0.0001
Depression	803	1.48	(1.38–1.59)	<0.0001	1.1	(1.02–1.18)	0.0131
Anxiety	1960	1.61	(1.53–1.69)	<0.0001	1.21	(1.15–1.27)	<0.0001
Alcoholism or alcohol-related disorders	57	0.97	(0.75–1.25)	0.8031	0.93	(0.72–1.21)	0.5941
Tobacco dependence	39	0.9	(0.66–1.23)	0.5098	0.83	(0.6–1.13)	0.2391
Obesity	109	1.34	(1.11–1.62)	0.0024	1	(0.83–1.21)	0.9629
Drugs used							
Oral steroids	7490	0.62	(0.6–0.64)	<0.0001	0.54	(0.52–0.56)	<0.0001
NSAIDs	11,804	0.21	(0.19–0.23)	<0.0001	0.24	(0.22–0.26)	<0.0001
Statins	2218	1.28	(1.22–1.34)	<0.0001	0.55	(0.52–0.58)	<0.0001

*NSAIDs* Nonsteroidal anti-inflammatory drugs

^a^ Crude HR represents relative hazard ratio

^b^ Adjusted HR represents adjusted hazard ratio mutually adjusted for accepted acupuncture, age, sex, diabetes mellitus, hypertension, hyperlipidemia, congestive heart failure, cerebrovascular diseases, depression, anxiety, alcoholism or alcohol-related disorders, tobacco dependence, obesity, oral steroids, NSAIDs, and statins in Cox proportional hazards regression

**Fig. 2:**
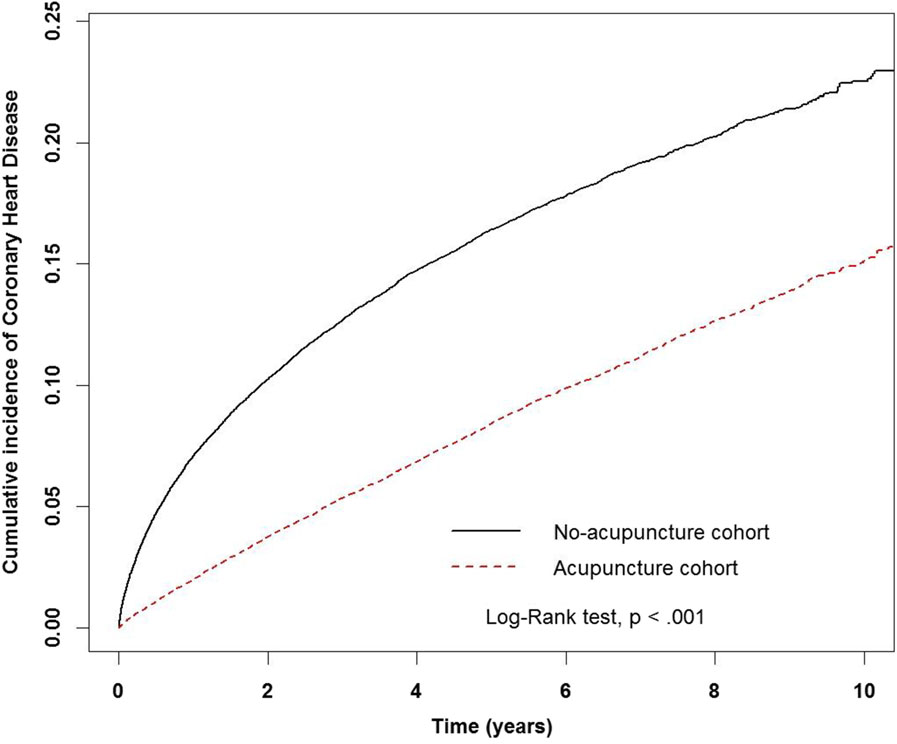
Cumulative incidence of coronary heart disease (CHD) between the acupuncture cohort and the no-acupuncture cohort. The cumulative incidence of CHD in the acupuncture cohort (*dashed line*) is significantly lower than in the no-acupuncture cohort (*solid line*) (log-rank test, *p* < 0.001)

Figure 2 reveals that the cumulative incidence of CHD was significantly lower in the acupuncture cohort during the follow-up period (log-rank test, *p* < 0.001). In this study, 4389 patients in the acupuncture cohort (17.44 per 1000 person-years) and 8133 patients in the no-acupuncture cohort (38.36 per 1000 person-years) developed CHD (adjusted HR 0.43, 95% CI 0.41–0.45) (Table 3). The beneficial effect of acupuncture on the incidence of CHD was observed in both female and male patients (adjusted HR 0.48 in women, 95% CI 0.46–0.51; adjusted HR 0.47 in men, 95% CI 0.45–0.50). Although the risk of CHD gradually increased with age, acupuncture significantly decreased the incidence of CHD in all age groups. Acupuncture decreased the risk of CHD in patients with fibromyalgia with or without comorbidities. Regardless of whether patients took oral steroids, NSAIDs, or statins, fewer patients in the acupuncture cohort developed CHD than in the no-acupuncture cohort.

**Table 3:** Incidence rates and hazard ratios for coronary heart disease among patients with fibromyalgia with or without acupuncture treatment

Variable	No acupuncture (*n* = 58,899)			Acupuncture (*n* = 58,899)				
	Events	Person-years	IR	Events	Person-years	IR	Crude HR (95% CI)	Adjusted HR^a^ (95% CI)
Total	8133	212,027	38.36	4389	251,711	17.44	0.48 (0.46–0.50)^b^	0.43 (0.41–0.45)^b^
Sex								
Female	4650	120,934	38.45	2488	141,235	17.62	0.48 (0.46–0.51)^b^	0.43 (0.41–0.45)^b^
Male	3483	91,093	38.24	1901	110,476	17.21	0.47 (0.45–0.5)^b^	0.45 (0.43–0.48)^b^
Age group, years								
18–39	877	92,941	9.44	371	94,477	3.93	0.42 (0.38–0.48)^b^	0.39 (0.35–0.44)^b^
40–59	5059	99,271	50.96	2887	133,190	21.68	0.45 (0.43–0.47)^b^	0.42 (0.41–0.44)^b^
≥60	2197	19,814	110.88	1131	24,044	47.04	0.48 (0.45–0.52)^b^	0.47 (0.43–0.5)^b^
Baseline comorbidities								
Diabetes mellitus								
No	6227	193,111	32.25	3301	225,893	14.61	0.47 (0.45–0.5)^b^	0.44 (0.42–0.46)^b^
Yes	1906	18,916	100.76	1088	25,818	42.14	0.46 (0.42–0.49)^b^	0.44 (0.41–0.47)^b^
Hypertension								
No	4148	174,292	23.80	2186	199,801	10.94	0.48 (0.45–0.5)^b^	0.43 (0.41–0.46)^b^
Yes	3985	37,735	105.61	2203	51,910	42.44	0.44 (0.42–0.47)^b^	0.44 (0.42–0.46)^b^
Hyperlipidemia								
No	5328	180,288	29.55	2742	210,271	13.04	0.46 (0.44–0.48)^b^	0.43 (0.41–0.45)^b^
Yes	2805	31,738	88.38	1647	41,440	39.74	0.49 (0.46–0.52)^b^	0.46 (0.43–0.49)^b^
Congestive heart failure								
No	7942	210,670	37.70	4282	249,749	17.15	0.48 (0.46–0.5)^b^	0.44 (0.42–0.45)^b^
Yes	191	1356	140.83	107	1963	54.52	0.45 (0.35–0.57)^b^	0.46 (0.36–0.59)^b^
Cerebrovascular diseases								
No	6867	199,807	34.37	3689	235,364	15.67	0.48 (0.46–0.5)^b^	0.44 (0.42–0.45)^b^
Yes	1266	12,220	103.60	700	16,348	42.82	0.46 (0.42–0.5)^b^	0.45 (0.41–0.49)^b^
Depression								
No	7628	203,237	37.53	4091	240,799	16.99	0.48 (0.46–0.49)^b^	0.44 (0.42–0.45)^b^
Yes	505	8790	57.45	298	10,912	27.31	0.51 (0.44–0.59)^b^	0.46 (0.4–0.53)^b^
Anxiety								
No	6860	191,626	35.80	3702	225,730	16.40	0.48 (0.46–0.5)^b^	0.44 (0.42–0.46)^b^
Yes	1273	20,401	62.40	687	25,982	26.44	0.46 (0.42–0.5)^b^	0.43 (0.39–0.47)^b^
Alcoholism or alcohol-related disorders								
No	8093	211,137	38.33	4372	250,521	17.45	0.48 (0.46–0.5)^b^	0.44 (0.42–0.46)^b^
Yes	40	889	44.99	17	1190	14.28	0.35 (0.2–0.61)^b^	0.33 (0.19–0.6)^b^
Tobacco dependence								
No	8109	211,341	38.37	4374	251,047	17.42	0.48 (0.46–0.5)^b^	0.44 (0.42–0.45)^b^
Yes	24	685	35.02	15	664	22.58	0.68 (0.35–1.29)	0.47 (0.23–0.94)^c^
Obesity								
No	8062	210,777	38.25	4351	250,115	17.40	0.48 (0.46–0.5)^b^	0.44 (0.42–0.46)^b^
Yes	71	1250	56.80	38	1596	23.81	0.45 (0.3–0.66)^b^	0.36 (0.24–0.55)^b^
Drugs used								
Oral steroids								
No	3458	56,042	61.70	1574	74,258	21.20	0.39 (0.37–0.41)^b^	0.39 (0.37–0.42)^b^
Yes	4675	155,985	29.97	2815	177,453	15.86	0.54 (0.52–0.57)^b^	0.49 (0.46–0.51)^b^
NSAIDs								
No	543	1878	289.11	175	3206	54.58	0.27 (0.23–0.32)^b^	0.31 (0.26–0.37)^b^
Yes	7590	210,148	36.12	4214	248,505	16.96	0.49 (0.47–0.51)^b^	0.45 (0.44–0.47)^b^
Statins								
No	6799	179,722	37.83	3505	215,745	16.25	0.46 (0.44–0.48)^b^	0.42 (0.4–0.44)^b^
Yes	1334	32,305	41.29	884	35,967	24.58	0.6 (0.55–0.66)^b^	0.56 (0.52–0.61)^b^

*IR* Incidence rate per 1000 person-years; NSAIDs Nonsteroidal anti-inflammatory drugs

^a^ Adjusted HR adjusted for accepted acupuncture, age, sex, diabetes mellitus, hypertension, hyperlipidemia, congestive heart failure, cerebral vascular diseases, depression, anxiety, alcoholism or alcohol-related disorders, tobacco dependence, obesity, oral steroids, NSAIDs, and statins in Cox proportional hazards regression

^b^ *p* < 0.001

^c^ *p* < 0.05

Table 4 reveals the top ten disease categories for which the patients with fibromyalgia in the acupuncture cohort received acupuncture treatment. The most common conditions leading to visiting acupuncture doctors were disorders of the musculoskeletal system and connective tissues that are highly related to fibromyalgia. Injury was also a common reason for receiving acupuncture.

**Table 4:** Top ten disease categories as reasons for clinical acupuncture visits in the acupuncture cohort

Disease categories (ICD-9-CM codes)	Acupuncture cohort (*n* = 58,899)
Disorders of musculoskeletal system and connective tissue (710–739)	42,488
Injury and poisoning (800–999)	36,465
Symptoms, signs, and ill-defined conditions (780–799)	4014
Disorders of nervous system (320–389)	2802
Disorders of digestive system (520–579)	1714
Disorders of respiratory system (460–519)	1497
Disorders of circulatory system (390–459)	850
Disorders of genitourinary system (580–629)	725
Disorders of skin and subcutaneous tissue (680–709)	335
Mental disorders (290–319)	283

*ICD-9-CM* International Classification of Diseases, Ninth Revision, Clinical Modification

More than one ICD-9-CM code can be recorded when patients receive acupuncture

## Discussion

To our knowledge, our present nationwide population-based study is the first to reveal that acupuncture decreases the risk of CHD in patients with fibromyalgia. As a popular treatment for fibromyalgia, acupuncture has been performed on patients with fibromyalgia in several clinical trials. However, the outcome evaluation was previously always focused on pain score, sleep quality, and QOL [10]. In our study, we found that the beneficial effects of acupuncture on developing CHD in patients with fibromyalgia were independent of sex, age, comorbidities, and anti-inflammatory drugs included in our data. Statins, the inhibitors of the 3-hydroxy-3-methylglutaryl-coenzyme A reductase for treating hyperlipidemia, were previously reported to reduce the risk of cardiovascular diseases [18]. Our study revealed that regardless of whether patients with fibromyalgia took statins, acupuncture lowered the risk of CHD.

Our study revealed that more than half of patients with fibromyalgia in Taiwan have received acupuncture. A previous report revealed that the leading reason for patients in Taiwan to seek acupuncture treatment was diseases of the musculoskeletal system and connective tissue [19]. Fibromyalgia also belongs to this disease category. This is true not only in Taiwan, because patients with fibromyalgia also receive acupuncture in the other countries, such as Germany [20]. Previous studies in the United States, Spain, and Iran have demonstrated the efficacy of acupuncture in fibromyalgia [21–23].

A strength of our study is the comprehensive, large-scale database that we used, Taiwan’s NHIRD. This database provides an enormous sample size to reduce selection and participation bias, and it includes long-term follow-up data [12]. In addition, the characteristics of patients with fibromyalgia in our study were consistent with those in previous reports of female and middle-aged predominance in other countries [6, 24]. These baseline characteristics were similar in both cohorts in our study.

In Taiwan, the majority of TCM doctors are graduated from baccalaureate TCM education programs (7- or 8-year medical doctor programs) or postbaccalaureate TCM programs (5-year medical doctor programs) in universities. The graduated students must pass the board examination conducted by the government. The training programs on the manipulation of acupuncture are standardized. The duration of needle retention most commonly performed is about 20–30 minutes in Taiwan, and achievement of deqi is a basic requirement in acupuncture treatment.

Pregabalin was the first FDA-approved drug for treatment of fibromyalgia [1]. It was demonstrated to improve pain, sleep disturbance, and fatigue in adult patients with fibromyalgia [25]. In our present study, we did not compare the efficacy of acupuncture with pregabalin, because this drug was not reimbursed by Taiwanese NHIA for fibromyalgia until 2015. Whether pregabalin decreases the incidence of CHD in patients with fibromyalgia has never been reported. Furthermore, pregabalin has cardiac adverse effects because it may induce heart failure [26]. Acupuncture has been reported to attenuate both ischemic injury of the heart and heart failure [27, 28]. Whether acupuncture treatment provides benefits to those patients receiving pregabalin deserves future investigation.

Fibromyalgia is thought to arise from aberrant brain chemistry, function, and structure [29]. The effects of acupuncture have also been documented to affect the central nervous system to ameliorate fibromyalgia [30]. An increasing body of evidence suggests that central sensitization contributes to hyperalgesia and allodynia in patients with fibromyalgia. Activation of *N*-methyl-d-aspartate receptor (NMDAR) and cyclic adenosine monophosphate-responsive element binding protein promotes hyperalgesia [29]. In a previous study on fibromyalgia, researchers using a murine model found that activation of the dorsal root ganglion was suppressed by acupuncture [31]. Activation of cardiac NMDAR induces oxidative stress and facilitates atrial fibrillation that were attenuated by NMDAR antagonists [32, 33]. Inhibition of NMDAR is the possible mechanism to prevent CHD in fibromyalgia, and whether acupuncture can suppress cardiac NMDAR in fibromyalgia needs further evaluation.

Other than the NMDAR pathway, the 5-HT_3_ receptor may be another target of acupuncture to decrease CHD in fibromyalgia. The analgesic effect of acupuncture is also evident through the 5HT_3_ receptor in a rat model of collagenase-induced arthritis, and 5-HT_3_ receptor antagonists have beneficial effects on autonomic cardiac dysfunction in patients with fibromyalgia [34, 35]. However, the results of our present study were contrary to those of a previous study involving transient receptor potential vanilloid 1 (TRPV1). Overexpression of TRPV1 in the spinal cord was suppressed by acupuncture in a fibromyalgia murine model [11], but TRPV1 in the endothelium prevents CHD [36]. The heart-protective effect of acupuncture in fibromyalgia may not be through the TRPV1 pathway.

Antidepression, anti-inflammation, and improvement of sleep quality are also possible mechanisms of acupuncture to prevent CHD in fibromyalgia. In previous reports, acupuncture improved sleep quality in patients with insomnia, which is highly associated with fibromyalgia and CHD [37–39]. Depression is a common associated symptom of fibromyalgia, and it increases the incidence of CHD through deregulation of the autonomic nervous system and the hypothalamic-pituitary-adrenal axis [40]. Acupuncture stabilized this axis, activated the hippocampal serotonin system, and suppressed inflammation to attenuate depression [41, 42].

Comorbidities of fibromyalgia, including DM, hypertension, and heart disease, are highly associated with chronic inflammation. Many previous studies of acupuncture were focused on the analgesic effect of acupuncture, but additional studies in recent years demonstrated that acupuncture attenuated inflammation. Acupuncture attenuated inflammation through the vagus nerve mediated by dopamine [43]. The combination of acupuncture and moxibustion relieved Crohn’s disease by decreasing the ratio of Th17 to Treg cells in the intestinal mucosa [44]. These studies support that acupuncture has not only an analgesic effect but also an anti-inflammatory effect.

In addition, researchers in a previous study found that acupuncture before percutaneous coronary intervention in patients with acute myocardial infarction attenuated cardiac injury [45]. Acupuncture also decreased myocardial infarct areas and preserved cardiac function through heat shock protein 20 (HSP20) and HSP27 in an animal study [27]. These reports demonstrated that acupuncture can protect the heart from ischemia. Future mechanistic study in this area is warranted.

Several limitations in our study are noted. First, the NHIRD did not provide the severity of fibromyalgia, including pain scores, the severity of inflammation, or daily life disabilities. To minimize the confounding factors, we performed a 1:1 propensity score match and found that the baseline characteristics of both cohorts were similar. The percentages of patients who used NSAIDs and oral steroids were also similar in both cohorts. The second limitation is that the NHIRD did not provide data about patients’ lifestyles, such as smoking, alcohol drinking, body mass index (BMI), stress, and exercise. Although detailed information about smoking exposure, alcohol drinking, and BMI was not available, we were able to acquire information on illnesses resulting from these personal habits and lifestyles. We conducted a 1:1 propensity score match that included the diagnoses of alcoholism or alcohol-related disorders, tobacco dependence, and obesity. Patients with these disorders were similar in both cohorts, and the protective effect of acupuncture on CHD was significant in patients with fibromyalgia with these comorbid diseases. However, there was still an underestimation of smoking exposure and alcohol drinking in both cohorts. On the basis of our findings in this matched cohort study, we have designed a prospective participant- and assessor-blinded, randomized controlled trial to investigate the efficacy and mechanisms of acupuncture in patients with fibromyalgia (ClinicalTrials.gov identifier: NCT02583334). These lifestyle factors are recorded using a patient-reported questionnaire and should be able to provide more information regarding lifestyle. The third limitation was that detailed information on selected acupoints was not provided in the dataset. Acupoint selection was individualized according to the TCM diagnosis of patients and the experience of doctors, which is more like an effectiveness study. Most studies on the efficacy of acupuncture fix the acupoints to standardize their treatment protocol. The clinical trial of acupuncture in patients with fibromyalgia we conducted also fixed the acupoints as LI4, LI11, LR3, ST36, SP6, and GB34, which are commonly used acupoints for the treatment of patients with fibromyalgia.

## Conclusions

Our present study confirms that acupuncture reduced the risk of CHD in patients with fibromyalgia in Taiwan. This noteworthy finding can provide some hints for further clinical and mechanistic studies.

## Abbreviations

BMI: Body mass index
CHD: Coronary heart disease
DM: Diabetes mellitus
FDA: U.S. Food and Drug Administration
HSP: Heat shock protein
ICD-9-CM: International Classification of Diseases, Ninth Revision, Clinical Modification
IR: Incidence rate
LHID 2000: Longitudinal Health Insurance Database 2000
NHI: National Health Insurance
NHIA: National Health Insurance Administration
NHIRD: National Health Insurance Research Database
NMDAR: *N*-methyl-d-aspartate receptor
NSAID: Nonsteroidal anti-inflammatory drug
QOL: Quality of life
TCM: Traditional Chinese medicine
TRPV1: Transient receptor potential vanilloid 1
